# The impact of different adverse childhood experiences on the dimensions of emotional dysregulation in adults with major depression

**DOI:** 10.3389/fpsyg.2025.1587042

**Published:** 2025-06-19

**Authors:** Paula Dagnino, Cecilia Cordeu, Eduardo Franco-Chalco, Sergio Gloger, Martín Duisallant, Joaquín Mizon, Loreto Romero

**Affiliations:** ^1^Facultad de Psicología y Humanidades, Universidad San Sebastián, Santiago, Chile; ^2^Escuela de Psicología, Universidad Bernardo O´Higgins, Santiago, Chile; ^3^Pontifical Catholic University of Peru, Lima, Peru; ^4^Facultad de Medicina, Universidad de Chile, Santiago, Chile; ^5^Private Practice, Santiago, Chile

**Keywords:** depression, childhood trauma, adverse childhood experiences, emotional dysregulation, adult patients

## Abstract

**Introduction:**

Adverse childhood experiences adversely affect the development of emotional regulation, yet their differential impact on discrete dysregulation dimensions in major depressive disorder remains underexamined. This study examines the relationship between adverse childhood experiences and emotional dysregulation, as well as its five dimensions.

**Methods:**

A total of 120 out-patients meeting the BDI-II cutoff for MDD completed the Childhood Trauma Questionnaire-SF (CTQ-SF) and the Difficulties in Emotion Regulation Scale (DERS). We first tested sex differences on DERS subscales (none emerged), then ran a multivariate multiple regression using Pillai’s trace to assess the joint effects of the five CTQ-SF dimensions on the five DERS dimensions. Six follow-up linear regressions predicted each DERS subscale and the total DERS score from the CTQ-SF dimensions.

**Results:**

Physical abuse was the only CTQ dimension with a significant multivariate effect. In univariate models, emotional abuse predicted higher overall dysregulation and increased emotional dyscontrol, everyday interference, and emotional inattention, whereas greater physical abuse was associated with reduced everyday interference.

**Discussion:**

Emotional abuse appears to be the principal driver of both global and facet-specific emotion-regulation difficulties in adults with MDD, suggesting that interventions emphasizing impulse control, emotion awareness, and reduction of functional interference may be particularly beneficial for this subgroup.

**Limitations:**

The cross-sectional, selfreport design precludes causal inferences and may be subject to recall bias; future work should employ longitudinal, multimethod approaches to elucidate mechanisms and resilience factors.

## Highlights

- ACEs affect emotional regulation, aligning with previous research. Particularly emotional abuse and physical abuse.
- Emotional abuse was shown to be linked with the dimensions of emotional dyscontrol, everyday interference, and emotional inattention.
- Physical abuse showed a better regulation of everyday interference.
- Therapy should focus on the management and control of emotions, toward the regulation of emotions to diminish their interference, and to attend to negative emotions affected by emotional abuse in childhood.

## 1 Introduction

Adverse childhood experiences (ACEs) are a significant public health issue, with recent systematic reviews indicating prevalence rates ranging from 12.5 to 50% globally (Madigan et al., 2023). These experiences, which include physical, emotional, and sexual abuse, as well as physical and emotional neglect, have been linked to a wide array of psychiatric disorders in adulthood, such as depression, anxiety, bipolar disorder, and personality disorders (Dagnino et al., 2020; Martínez et al., 2024; Martins et al., 2011; Sahle et al., 2022; Li et al., 2020). The theoretical framework of attachment theory provides a lens through which the impact of ACEs on emotional regulation can be understood. According to Bowlby (1969), early interactions with caregivers form the basis of internal working models that influence emotional processing and regulation throughout life. Disruptions in these early relationships, such as those caused by abuse or neglect, can lead to insecure attachment patterns, which are associated with difficulties in emotional regulation (Mikulincer and Shaver, 2019).

The relationship between ACEs and emotional dysregulation has been the subject of extensive research. Emotion regulation is a multifaceted construct that is essential for adaptive psychological functioning. It involves different dimensions, such as awareness, understanding, and acceptance of emotions, as well as the ability to control impulsive behaviors and act in accordance with desired goals when experiencing intense emotions (Aldao et al., 2010; Cole et al., 1994; Gratz et al., 2016; Gross, 1998). Emotional dysregulation, characterized by deficits in these processes, is increasingly recognized as a transdiagnostic factor across various psychopathologies (Iwakabe et al., 2023; Sloan et al., 2017; Weissman et al., 2019) and, when originating in childhood from ACEs can contribute to the development, maintenance, and treatment of many psychiatric disorders, including depression (Berking and Wupperman, 2012; Gross and Muñoz, 1995; Kring and Werner, 2004). Taking into account its importance, various therapeutic approaches have rightly placed the focus of treatment on developing the capacity for emotional regulation (Barlow et al., 2016; Berking and Wupperman, 2012; Heard and Linehan, 1994; Lew-Starowicz et al., 2020; McMain et al., 2010; Panagou and MacBeth, 2022).

Several studies have shown that early exposure to abuse and neglect can alter emotional development, leading to difficulties in emotion regulation in adulthood (Dagnino et al., 2020; Gloger et al., 2021; Palmier-Claus et al., 2025; Warmingham et al., 2023). Some studies have provided empirical evidence of the relationship between particular ACEs and difficulties in emotion regulation. For example, girls who suffered sexual abuse had difficulty understanding and regulating emotions, and neglected children have been shown to be less able to understand negative emotions and have fewer regulating skills (Shipman et al., 2000; Shipman et al., 2005). Among the common types of childhood adversity, emotional abuse, in particular, has been identified as a potent predictor of emotional dysregulation, with studies indicating that it has a more substantial impact on regulatory difficulties than other forms of maltreatment (Huh et al., 2017; Humphreys et al., 2020; Li et al., 2021).

Despite the established link between ACEs and emotional dysregulation, research on how specific types of ACEs might differentially affect specific dimensions of emotional dysregulation remains limited. Emotional dysregulation can be broken down into five dimensions: emotional dyscontrol, inattention, confusion, rejection, and daily interference (Gratz and Roemer, 2004). Cheng and Langevin (2023) found that emotional abuse increased the sensibility toward the recognition of emotions such as anger, fear, and sadness. On the other hand, they found that physical neglect was related to impulsive, out-of-control behavior and task-oriented interference, and physical abuse was associated with more significant inattention to emotions, such as fear.

The present study seeks to contribute to the existing literature by examining the relationship between different types of ACEs—physical, emotional, and sexual abuse, as well as physical and emotional neglect—and specific dimensions of emotional dysregulation—emotional dyscontrol, inattention, confusion, rejection, and daily interference—in a clinical sample of patients with major depressive disorder (MDD). Based on existing literature, we hypothesize that emotional abuse will show strong associations with regulatory difficulties. Additionally, we aim to explore the specific patterns of association between different types of ACEs and the dimensions of emotional dysregulation, contributing to a more nuanced understanding of these relationships.

By situating this study within the broader context of ACE research and emotional regulation theory, we aim to provide a comprehensive analysis of how early adverse experiences shape emotional functioning in adulthood. This research has the potential to inform therapeutic interventions by highlighting the need to address specific regulatory deficits associated with different types of ACEs, ultimately improving outcomes for individuals with a history of childhood adversity.

## 2 Methods

### 2.1 Participants and procedure

Secondary data from 120 patients who consulted psychotherapy during 2018 and 2019 were analyzed. The original sample was collected by the lead author during her Fondecyt project N°11,170,561 with the approval of the ethics committee of the Universidad Alberto Hurtado according to Helsinky’s statement. For the original study, the inclusion criterion was patients over 18 years of age who consulted for psychological care at two outpatient centers in the city of Santiago. Patients who presented with Major Depression according to the cutoff point of the Chilean Beck Depression Scale (BDI-II, Beck Depression Inventory; Beck et al., 1961, BDI > 13) were included. Patients with psychotic disorders, addictions, eating disorders, or cognitive alterations were excluded because they required multidisciplinary and specialized care.

The procedure consisted of field assistants inviting patients scheduled for their first session with psychologists in two care centers to participate. The patients were clinically diagnosed with depression by a general practitioner or a psychiatrist. If the patient accepted and after reading and signing the consent form, the BDI was administered, and if they met the criteria, the rest of the paper instruments were given to the patients to fill out. The assistant remained present if there were any doubts, and the patient was identified with a code.

The sample comprised 70.83% of women, with a mean age of 35.61 years (SD = 13.14). This gender distribution is consistent with epidemiological data indicating that major depressive disorder is more prevalent among women than men (Kuehner, 2017; World Health Organization, 2017). Additionally, women are generally more likely to seek psychological help which may have contributed to the higher proportion of female participants in our sample. In terms of occupation, 14% of the participants reported being a homeowner, 22% of the participants reported being students, while most of the participants (49%) reported being dependent workers; the self-employed, unemployed, and those on medical leave represented the remaining 15% of the sample. On the other hand, 54% of the respondents reported that they were without a partner and 46% were couples. Participants had a mean of 27 (SD = 9.2) on the BDI, which is considered as Major Depressive disorder (MDD; Valdés et al., 2017).

### 2.2 Measurements

#### 2.2.1 Adverse childhood experiences (CTQ-SF)

The short form of the Childhood Trauma Questionnaire (CTQ-SF; Bernstein et al., 1994), which identifies the history of adversity in adults, was used to assess ACE. It is a 28-item self-report instrument in which people respond to certain conditions and/or experiences that occurred to them during childhood in a retrospective manner. It consists of five types of child maltreatment (physical, emotional and sexual abuse, and physical and emotional neglect), as well as a three-item scale to detect cases of underestimation of maltreatment. The latter was not used for the analysis. For each item, the subject responded on a 5-point Likert scale from 1 “never” to 5 “almost always.” Dimension scores were computed by averaging the raw scores of the items corresponding to each subscale, rather than summing them. This approach results in subscale scores that can include decimal values and remain on the original Likert scale range (1 to 5), facilitating interpretation and comparability across dimensions. This instrument was validated by Behn et al. (2020) in Chile. Cronbach’s *α* coefficients for the subscales of the Chilean version of the CTQ-SF were emotional abuse (0.85), physical abuse (0.87), sexual abuse (0.93), emotional neglect (0.79), and physical neglect (0.41). In the present study the Cornbach’s α coefficients for the subscales were: emotional abuse (0.86), physical abuse (0.83), sexual abuse (0.91), emotional neglect (0.86), and physical neglect (0.63).

#### 2.2.2 Emotional dysregulation (DERS)

The Difficulties in Emotion Regulation Scale (DERS) by Gratz and Roemer (2004), a 36item questionnaire that evaluates the difficulties adults face in emotional regulation, was used to assess emotional dysregulation. The items were grouped into five scales: emotional dyscontrol, inattention, confusion, emotional rejection, and daily interference, together with a total score. Example: (1) Emotional dyscontrol (e.g., “When I get angry, I lose control over my behaviors”); (2) Emotional inattention (e.g., “I am attentive to my feelings”); (3) Emotional confusion (e.g., “I feel confused about how I feel”); (4) Emotional rejection (e.g., “I do not tolerate when I get angry”); (5) Daily interference (e.g., “When I get upset, I have a hard time concentrating”).

The items are scored on a 5-point scale ranging from 1 (“almost never” to 5 “almost always”). The instrument was validated by Guzmán-González et al. (2014), with good internal consistency indices (*α* = 0.66 and α = 0.89) similar to or even higher than its Spanish version (Hervás and Jódar, 2008). In the present study, the general scale showed a Cronbach’s α coefficient of 0.94, indicating an excellent internal consistency. As for the previous scale, dimension scores were also computed by averaging the raw scores of the items.

### 2.3 Data analysis

To address the objectives of the present study, a Multivariate Multiple Regression Model was conducted to examine the associations between adverse childhood experiences, as measured by the dimensions of the Childhood Trauma Questionnaire (CTQ), and difficulties in emotion regulation, as assessed by the dimensions of the Difficulties in Emotion Regulation Scale (DERS). The multivariate test of model significance was evaluated using Pillai’s trace, which is considered robust to violations of homogeneity of variance–covariance matrices and offers a conservative estimation of the multivariate effect. Before conducting the regression analyses, sex differences in the dependent variables (DERS subscales) were evaluated due to a higher proportion of women in the sample. Independent sample t-tests revealed no statistically significant differences between men and women on any DERS subscales. Therefore, sex was not a control variable in the final analyses. Following the multivariate model, six multiple regression models were estimated, each predicting a separate DERS subscale or the total DERS score. The predictor variables for all models were the five dimensions of the CTQ (emotional abuse, physical abuse, sexual abuse, emotional neglect, and physical neglect). This allowed for a more detailed understanding of how different forms of childhood adversity relate to specific difficulties in emotional regulation. Assumptions of the regression models were evaluated before interpretation. The normality and homoscedasticity of residuals were tested using the Shapiro–Wilk and Breusch–Pagan tests, respectively, and both assumptions were met for all models. Multicollinearity was assessed through the Variance Inflation Factor (VIF); the highest VIF observed was for emotional negligence (2.11), while all the other VIF values fell below the commonly accepted threshold of 5 (Belsley et al., 1980). Influential cases were examined using Cook’s distance, and no value exceeded 1, indicating the absence of highly influential observations. All statistical analyses were conducted using R software (R Core Team, 2022).

## 3 Results

The descriptive results indicate that among the adverse childhood experiences (CTQ), emotional abuse and emotional neglect had the highest average scores, suggesting they were the most frequently reported forms of adversity in this sample. In contrast, physical and sexual abuse, along with physical neglect, showed lower average values, indicating less frequent endorsement. For the emotion regulation difficulties (DERS), everyday interference and emotional rejection presented the highest mean scores, suggesting these were the most prominent challenges experienced by participants. In contrast, emotional inattention showed the lowest mean, followed by emotional dyscontrol and confusion, indicating moderate difficulty levels in these areas. Overall, the global DERS score was moderate (M = 3.04, SD = 0.85), suggesting a general but not severe presence of emotion regulation difficulties in the sample (Table 1).

**Table 1:** Descriptive statistics for the ACEs and DERS reported by the study participants.

Variable	Mean	SD	Median	Min	Max
CTQ					
Emotional abuse	2.53	1.18	2.40	0.80	5.00
Physical abuse	1.66	0.86	1.30	0.80	4.20
Sexual abuse	1.59	0.97	1.00	0.40	5.00
Emotional negligence	2.26	1.01	2.10	1.00	4.60
Physical negligence	1.49	0.52	1.40	0.80	3.40
DERS					
Emotional rejection	3.30	1.23	3.29	1.00	5.00
Everyday interference	3.77	1.07	4.00	1.00	5.00
Emotional inattention	2.64	0.94	2.40	1.00	4.80
Emotional dyscontrol	2.74	1.27	2.50	1.00	5.00
Emotional confusion	2.76	1.11	2.67	1.00	5.00
Global Score	3.04	0.85	2.96	1.28	4.80

The multivariate multiple regression analysis, shown in Table 2, revealed that among the dimensions of childhood adversity, physical abuse was the only predictor that showed statistically significant multivariate effects on the set of emotion regulation difficulties [Pillai’s Trace = 0.11, *F*(5, 114) = 2.69, *p* = 0.025, η^2^ = 0.11]. In contrast, emotional abuse, sexual abuse, emotional neglect, and physical neglect did not show significant multivariate effects, with all *p*-values well above the 0.05 threshold.

**Table 2:** Multivariate multiple regression model results of adverse childhood experiences on difficulties in emotional regulation.

ACE	DF	Pillai’s trace	F	*p*	η^2^
Emotional abuse	5	0.09	2.12	0.068	0.09
**Physical abuse**	**5**	**0.11**	**2.69**	**0.025**	**0.11**
Sexual abuse	5	0.02	0.49	0.780	0.02
Emotional negligence	5	0.01	0.40	0.849	0.02
Physical negligence	5	0.03	0.68	0.640	0.03
Residuals	110				

Values in bold indicate statistically significant effects.

Subsequent multiple regression analyses (Table 3) were performed to further delineate the associations between specific ACEs and each DERS dimension, including the overall DERS score. The regression analysis predicting the total DERS score revealed that emotional abuse exerted a positive, moderate, and statistically significant effect (b = 1.32, SE = 0.53, *p* = 0.014, standardized coefficient B = 0.37, 95% CI [0.07, 0.66]), indicating that higher levels of reported emotional abuse were associated with more significant overall emotional dysregulation.

**Table 3:** Regression models of adverse childhood experiences on difficulties in emotional regulation subscales and global score.

Variable	Global score		Emotional dyscontrol		Everyday interference		Emotional rejection		Emotional inattention		Emotional confusion	
	*b (SE)*	*B [95% CI]*	*b (SE)*	*B [95% CI]*	*b (SE)*	*B [95% CI]*	*b (SE)*	*B [95% CI]*	*b (SE)*	*B [95% CI]*	*b (SE)*	*B [95% CI]*
Intercept	**64.16 (6.27)**	**–**	**17.3 (2.49)**	**–**	**14.23 (1.29)**	**–**	**12.34 (1.46)**	**–**	**13.33 (2.28)**	**–**	**6.95 (1.02)**	–
Emotional abuse	**1.32 (0.53)**	**0.37 [0.07–0.66]**	**0.65 (0.21)**	**0.45 [0.16–0.73]**	**0.24 (0.11)**	**0.32 [0.02–0.62]**	−0.05 (0.12)	−0.06 [−0.37–0.25]	**0.41 (0.19)**	**0.32 [0.02–0.62]**	0.07 (0.08)	0.12 [−0.18–0.43]
Physical abuse	−0.65 (0.53)	−0.13 [−0.35–0.08]	−0.39 (0.21)	−0.19 [−0.40–0.02]	**−0.27 (0.11)**	**−0.27 [−0.49 - -0.05]**	0.02 (0.13)	0.02 [−0.21–0.25]	−0.08 (0.2)	−0.04 [−0.26–0.18]	0.06 (0.09)	0.08 [−0.15–0.30]
Sexual abuse	0.35 (0.46)	0.09 [−0.13–0.29]	0.10 (0.18)	0.06 [−0.15–0.26]	0.02 (0.1)	0.02 [− 0.19–0.24]	0.1 (0.11)	0.1 [−0.12–0.32]	0.14 (0.17)	0.09 [−0.12–0.30]	−0.01 (0.08)	−0.01 [−0.23–0.20]
Emotional negligence	−0.05 (0.54)	−0.01 [−0.27–0.24]	−0.07 (0.21)	−0.04 [−0.29–0.21]	−0.08 (0.11)	−0.09 [−0.35–0.17]	0.12 (0.13)	0.13 [−0.14–0.40]	−0.08 (0.2)	−0.05 [−0.31–0.20]	0.05 (0.09)	0.08 [−0.18–0.34]
Physical negligence	−0.19 (0.84)	−0.02 [−0.22–0.18]	0.11 (0.33)	0.03 [−0.16–0.23]	0.11 (0.17)	0.07 [−0.14–0.27]	−0.11 (0.2)	−0.06 [−0.28–0.15]	−0.23 (0.3)	−0.08 [−0.28–0.13]	−0.08 (0.13)	−0.06 [−0.27–0.15]
R2		0.08		0.12		0.04		0.01		0.05		0.003

b, Non-standardized coefficient; SE, Standard error; B, Standardized coefficients; CI, Confidence interval. Bold coefficients are significant.

For the emotional dyscontrol subscale, emotional abuse was again a significant predictor (b = 0.65, SE = 0.21, *p* = 0.002, B = 0.45, 95% CI [0.16, 0.73]), suggesting that increased emotional abuse corresponds with elevated dyscontrol. Regarding everyday interference, the analyses showed a significant positive effect of emotional abuse (b = 0.24, SE = 0.11, *p* = 0.034, B = 0.32, 95% CI [0.02, 0.62]) and a significant negative effect of physical abuse (b = −0.27, SE = 0.11, *p* = 0.017, B = −0.27, 95% CI [−0.49, −0.05]), implying that while emotional abuse increases daily interference, higher reports of physical abuse are associated with lower interference levels. For the emotional inattention subscale, emotional abuse significantly predicted higher scores (b = 0.41, SE = 0.19, *p* = 0.033, B = 0.32, 95% CI [0.02, 0.62]), whereas the other ACE variables did not reach significance. Finally, the regression models for the emotional rejection and emotional confusion subscales did not reveal any significant effects from the ACE predictors (all *p* > 0.05).

## 4 Discussion

Prior research has investigated the harmful effects of adverse childhood experiences on regulating emotions in adulthood. This study aimed to examine the relationship between different types of ACEs—physical, emotional, and sexual abuse, as well as physical and emotional neglect—and specific dimensions of emotional dysregulation—emotional dyscontrol, inattention, confusion, rejection, and daily interference—in a clinical sample of adult patients with major depressive disorder.

In this study, patients reporting adverse experiences in childhood show an association with emotion dysregulation. This finding goes in line with the large number of studies that have highlighted the relationship between adverse experiences leading to more significant difficulties in interpreting, managing, and processing emotions in adulthood (Brassard et al., 2000; Cheng and Langevin, 2023; Dye, 2020; Gibb et al., 2009; Green, 1988; Heim et al., 2004; Kim et al., 2023; Medeiros Assed et al., 2020; Seitz et al., 2021; Shipman and Zeman, 1999; Warmingham et al., 2019; Young and Widom, 2014; Young and Widom, 2014). Several theories that address emotion regulation development, mainly attachment theory, state that an abusive or neglectful relationship between the child and his caregiver would lead to increased insecurity, avoidance, and isolation which leads to a context that invalidates emotions (Bowlby, 1969; Li et al., 2021; Reeves, 2008). Erkoreka et al. (2022) highlight the central role of insecure attachment in childhood adversity, emotional regulation, and their relationship.

In accordance with our hypothesis, emotional abuse showed a positive association with emotional dysregulation. Emotional abuse refers to caregivers making hurtful comments and/or making a child feel unwanted. Emotional abuse has been considered a more “silent” form of maltreatment (Hart and Brassard, 1987; Hibbard et al., 2012). It directly targets emotional and cognitive processes, disrupts attachment relationships, creates chronic emotional arousal and leads to negative-self perceptions and self-criticism (Heleniak et al., 2016; Spinazzola et al., 2014). This seems to be in the background of other types of abuse (e.g., sexual abuse), which may lead to emotional dysregulation through trauma-related mechanisms, or neglect, which involves passive absence of care (O’Mahen et al., 2015). Following this idea, it makes sense that individuals living in an emotionally abusive environment feel invalidated (their emotions are wrong or unacceptable) and creates a state of chronic emotional arousal using maladaptive strategies to survive (Cheng and Langevin, 2023).

The second adverse childhood experience that showed a positive association with emotional dysregulation is physical abuse. This finding aligns with the work of Unger and De Luca (2014), which demonstrated that adults who were physically abused as children are more likely to exhibit fearful and dismissing avoidant attachment styles. These attachment styles, characterized by difficulty trusting others and managing emotional closeness, may stem from the chronic stress and fear experienced during childhood (Heim and Nemeroff, 2001). Physical abuse often disrupts the development of a secure attachment bond with caregivers, as the caregiver—who should ideally provide safety and comfort—becomes a source of fear and harm.

Our second aim was to expand these findings by exploring which of the dimensions that make up emotional dysregulation (emotional dyscontrol, inattention, confusion, rejection, and daily interference) are related to each adverse experience (emotional, physical, sexual abuse, and physical and emotional neglect). The results showed that emotional abuse was associated with three subdimensions: emotional dyscontrol (difficulties in controlling impulses when faced with adverse situations), everyday interference (challenges in engaging in goal-directed behaviors when experiencing negative emotions), and emotional inattention (lack of awareness of emotional responses).

Emotional dyscontrol refers to difficulties in controlling impulses when faced with adverse situations. This is often linked to heightened emotional reactivity and impaired inhibitory control caused by emotional abuse (Christ et al., 2019). Patients who experienced emotional abuse also showed difficulties engaging in goal-directed behavior through having problems focusing and maintaining motivation under stress, as it undermines self-efficacy and fosters maladaptive coping mechanisms (Gratz and Roemer, 2004). Finally, the association with a lack of awareness of emotional responses (emotional inattention) suggests that emotional abuse results from a continuous invalidation of emotional experiences during childhood, leading individuals to suppress or disconnect from their emotions as a defense mechanism (Cheng and Langevin, 2023).

The other adverse childhood experience was physical abuse, which was associated negatively with everyday interference. Patients who reported having high physical abuse regulated better when engaging in goal-directed behaviors in negative emotional contexts. This association was unexpected. One possible hypothesis is that, in some cases, exposure to physical abuse may foster adaptive, goal-directed behaviors as survival strategies (Dvir et al., 2014). However, the relationship between adversity and resilience is complex and varies across individuals (Afifi and MacMillan, 2011). In contrast, emotional abuse appears more consistently linked to emotional dysregulation, possibly due to chronic invalidation and erosion of self-worth (Christ et al., 2019). These interpretations are exploratory and should be viewed as hypotheses for future research. Further studies are needed to clarify the mechanisms underlying these associations.

Overall, these findings have significant implications for clinical practice and for the design of therapeutic interventions aimed at individuals who have experienced adverse childhood experiences. Early identification of those who have suffered emotional abuse and the implementation of appropriate intervention programs could help mitigate the long-term adverse effects of emotional regulation and improve the quality of life of these individuals. As Panagou and MacBeth (2022) refer, interventions should target the development of emotional regulation. Therefore, therapies that focus on improving emotional awareness (e.g., mentalization based approach; Wagner-Skacel et al., 2022, mindfulness-based therapies; Kabat-Zinn, 2003), impulse control (e.g., dialectical behavior therapy; Hall et al., 2021), and goal-directed behavior (e.g., cognitive-behavioral therapy; Beck, 1979) may be particularly beneficial for individuals with a history of emotional abuse and can promote better emotional and interpersonal functioning. These strategies highlight the importance of addressing emotional regulation deficits as a pathway to improving mental health outcomes in individuals affected by adverse childhood experiences.

### 4.1 Limitations of the study

This study has several limitations. First, this was a cross-sectional study that assessed measurements concurrently. To establish causal relationships, future longitudinal studies following children experiencing early life trauma are necessary. Second, all participants were patients with major depressive disorder, mainly women; there was no non-clinical sample, so the results obtained cannot be generalized. It is advisable to diversify the sample in future studies to cover different clinical profiles (of diverse pathology) and a non-clinical sample. Third, the three measures were assessed with self-reports. Many patients may have distorted mental representations of their emotion regulation ability and memories of adverse experiences due to their depression. Therefore, interpretation of the related results should be cautious. Fourth, other factors such as resilience factors (e.g., Chandler et al., 2015), perceived social support (e.g., Giovanelli et al., 2020; Kealy et al., 2020), and/or personality functioning (Dagnino et al., 2020) were not evaluated. All of them can influence positively or negatively the impact of adversity in childhood with emotion regulation in adulthood. Finally, future research suggests considering the diversity of types of abuse and trauma that occur today and in different settings (school, home, community, etc.), which do not fit the classic definitions of adverse childhood experiences (Finkelhor et al., 2015; Finkelhor, 2018) using this and other instruments will complement and amplify the study of the subject.

## 5 Conclusion

Despite its limitations, this study provides evidence that adverse childhood experiences have a significant impact on emotion dysregulation in a clinical sample of patients with major depressive disorder. Emotional abuse was found to be the most influential factor in emotional dysregulation. Specifically, it relates to emotional dyscontrol, everyday interference, and emotional inattention. This suggests that therapeutic interventions must be personalized for patients who have experienced this kind of abuse, targeting these specific dysregulations and aiming to develop more adaptative strategies.

## Data Availability

The data analyzed in this study is subject to the following licenses/restrictions: the data set used in this study are available from the corresponding authors upon request. Requests to access these datasets should be directed to Paula Dagnino, paula.dagnino@uss.cl.
