# Supplementary material for: The impact of different adverse childhood experiences on the dimensions of emotional dysregulation in adults with major depression
Source: Front Psychol. 2025 Jun 19;16:1587042. doi: 10.3389/fpsyg.2025.1587042 (PMC12231488; doi:10.3389/fpsyg.2025.1587042)
Supplement: Supplementary file 1 [file Table_1.docx]

**Correlation Matrix for the CTQ and DERS subscales**

|  | 1 | 2 | 3 | 4 | 5 | 6 | 7 | 8 | 9 | 10 |
| --- | --- | --- | --- | --- | --- | --- | --- | --- | --- | --- |
| 1. CTQ: Emotional abuse |  |  |  |  |  |  |  |  |  |  |
| 2. CTQ: Physical abuse | 0.57*** |  |  |  |  |  |  |  |  |  |
| 3. CTQ: Sexual abuse | 0.53*** | 0.23* |  |  |  |  |  |  |  |  |
| 4. CTQ: Emotional negligence | 0.70*** | 0.45*** | 0.36*** |  |  |  |  |  |  |  |
| 5. CTQ: Physical negligence | 0.43*** | 0.34*** | 0.26* | 0.47*** |  |  |  |  |  |  |
| 6. DERS: Emotional rejection | 0.35*** | 0.07 | 0.24* | 0.22* | 0.15 |  |  |  |  |  |
| 7. DERS: Everyday interference | 0.15 | -0.10 | 0.12 | 0.05 | 0.08 | 0.53*** |  |  |  |  |
| 8. DERS: Emotional inattention | 0.07 | 0.05 | 0.10 | 0.1 | 0.00 | 0.17 | 0.11 |  |  |  |
| 9. DERS: Emotional dyscontrol | 0.27* | 0.11 | 0.21* | 0.15 | 0.04 | 0.64*** | 0.58*** | 0.15 |  |  |
| 10. DERS: Emotional confusion | 0.19* | 0.16 | 0.08 | 0.17 | 0.05 | 0.45*** | 0.43*** | 0.49*** | 0.47*** |  |
| 11. DERS: Global Score | 0.31*** | 0.08 | 0.23* | 0.2* | 0.1 | 0.85*** | 0.72*** | 0.44*** | 0.84*** | 0.7*** |
| * p < 0.05, ** p < 0.01, *** p < 0.001 | | | | | | | | | | |
